# Evaluation and improvements of clustering algorithms for detecting remote homologous protein families

**DOI:** 10.1186/s12859-014-0445-4

**Published:** 2015-02-05

**Authors:** Juliana S Bernardes, Fabio RJ Vieira, Lygia MM Costa, Gerson Zaverucha

**Affiliations:** 1 Programa de Engenharia de Sistemas e Computação, COPPE, Universidade Federal do Rio de Janeiro, Rio de Janeiro, Brazil; 2 Engenharia de Computação e Informação, Universidade Federal do Rio de Janeiro, Rio de Janeiro, Brazil; 3 Sorbonne Universités, UPMC Univ Paris 06, UMR 7238, Biologie Computationnelle et Quantitative, Paris, France

**Keywords:** Sequence analysis, Clustering sequence algorithms, Remote homology detection

## Abstract

**Background:**

An important problem in computational biology is the automatic detection of protein families (groups of homologous sequences). Clustering sequences into families is at the heart of most comparative studies dealing with protein evolution, structure, and function. Many methods have been developed for this task, and they perform reasonably well (over 0.88 of F-measure) when grouping proteins with high sequence identity. However, for highly diverged proteins the performance of these methods can be much lower, mainly because a common evolutionary origin is not deduced directly from sequence similarity. To the best of our knowledge, a systematic evaluation of clustering methods over distant homologous proteins is still lacking.

**Results:**

We performed a comparative assessment of four clustering algorithms: Markov Clustering (MCL), Transitive Clustering (TransClust), Spectral Clustering of Protein Sequences (SCPS), and High-Fidelity clustering of protein sequences (HiFix), considering several datasets with different levels of sequence similarity. Two types of similarity measures, required by the clustering sequence methods, were used to evaluate the performance of the algorithms: the standard measure obtained from sequence–sequence comparisons, and a novel measure based on profile-profile comparisons, used here for the first time.

**Conclusions:**

The results reveal low clustering performance for the highly divergent datasets when the standard measure was used. However, the novel measure based on profile-profile comparisons substantially improved the performance of the four methods, especially when very low sequence identity datasets were evaluated. We also performed a parameter optimization step to determine the best configuration for each clustering method. We found that TransClust clearly outperformed the other methods for most datasets. This work also provides guidelines for the practical application of clustering sequence methods aimed at detecting accurately groups of related protein sequences.

## Background

Protein family detection is of fundamental importance in structural and functional genomics. Well-characterized protein families can contribute significantly to the delineation of functional diversity of homologous proteins, providing valuable evolutionary insights. In general, a protein family comprises a group of proteins that possess similar or identical functions, indicating that they were derived from a common ancestor and probably share important properties such as tertiary structure, functional sites, and interaction patterns. A protein family can be detected automatically by clustering methods that group together related proteins. These approaches partition data into groups, such that proteins in the same group are similar and proteins in different groups are dissimilar to each other. To detect protein families, clustering algorithms should take into account all similarity relationships in a given set of sequences. For this purpose, it is usual to carry out an all-against-all sequence–sequence similarity search using the BLAST program [1] to construct a similarity matrix that is then used by the clustering method to form protein groups.

**Figure 1:**
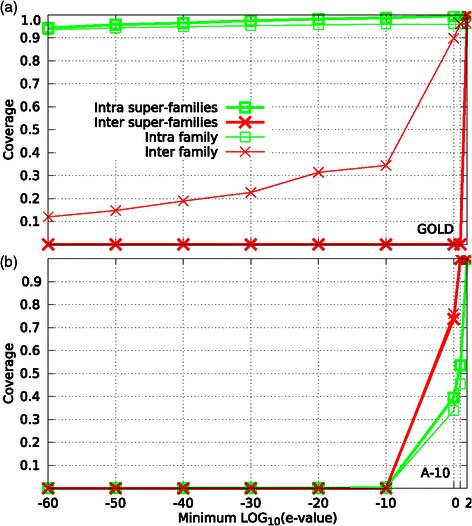
**Distribution of minimum BLAST e-values for GOLD and ASTRAL A-10 datasets.** The GOLD dataset **(a)** is a collection of enzymes that were manually assigned to protein families/super-families. A-10 **(b)** is an ASTRAL subset of the SCOP database that contains only sequences with identities less than 10%. For each protein in both datasets, we considered the e-value to the nearest neighbor from its own family/superfamily (intra curves) and the e-value to the nearest neighbor from any other family/superfamily (inter curves).

A number of clustering methods have been proposed to detect protein families, but to the best of our knowledge, the performance of most of them have been evaluated only on datasets containing homologous sequences with high identity. For instance, the GOLD dataset [2], which contains enzymes that were assigned manually to protein families, is often used to evaluate the performance of clustering methods. We argue that this benchmark is a relatively less complex case for clustering methods because, in general, members of the same protein family or super-family (groups of related families) are closer to each other than to any other family/super-family, as showed in Figure 1a. Conversely, when we used the SCOP dataset [3], a reference database that is used to study distantly related homologous proteins, we found that it was much more difficult to group the homologous proteins with very low sequence identity, as shown in Figure 1b where the curves are inverted compared with the curves in Figure 1a. This finding shows that members of the same family are so distant that members of different families seem to be closer to each other. Thus, the existing clustering methods yield adequate results for close homologs, but they are likely to fail in identifying distant evolutionary relatedness.

Here, we performed an extensive evaluation of clustering methods on distantly related homologous proteins to determine whether sequence-clustering algorithms can effectively detect remote homologous protein families. We evaluate four state-of-the-art methods: Markov Clustering (MCL) [4], Transitive Clustering (TransClust) [5], Spectral Clustering of Protein Sequences (SCPS) [6] and High-Fidelity clustering of sequences (HiFix) [7]. These four methods were assessed on various datasets with different level of difficulty (i.e., the datasets represent different sequence identity percentages) and on two clustering scenarios (i.e., family and super-family). The parameters were varied for each algorithm to find the best results of the clustering methods. We also determined whether the performance of sequence clustering methods could be improved using a profile-profile search instead of the traditional pairwise sequence search. We used HHblits [8] to build a profile for each sequence in the datasets, and we compared pairs of profiles instead of pairs of sequences to provide a similarity measure for each protein pair.

Our results show that the traditional similarity measure based on sequence–sequence comparisons, which is often used to feed sequence-clustering methods, is not suitable for detecting remote homologous protein families and super-families. We found that the clustering performance can be improved considerably by replacing the BLAST similarity measure with a novel measure based on profile-profile comparisons. We highlight that profiles can properly represent conserved evolutionary properties and they can be used to produce an insightful distance mesure for clustering methods. This novel measure increased the clustering performance of all methods across all datasets tested.

## Results and discussion

We assessed the performance of four clustering methods on eight different datasets by considering two clustering scenarios (family and super-family). Two types of similarity matrices were used to evaluate the performance of the algorithms. The first matrix was based on sequence-sequence comparisons obtained from BLAST (Section ‘Sequence–sequence comparisons’).

The second matrix was based on profile-profile comparisons (performed by HHblits and HHsearch), which was developed and used here for the first time (Section ‘Profile-profile comparisons’). To obtain the best possible results for each method, we performed a parameter optimization step to obtain the ideal set of parameters for each clustering algorithm (Section ‘Parameter optimization’). Finally, we discuss how the methodology proposed here could be employed to improve the results in practical applications (Section ‘Practical usage’).

### Sequence–sequence comparisons

We constructed a similarity matrix by extracting e-values from BLAST, named **S**equence **S**equence **C**omparisons (SSCs), see Section ‘Sequence–sequence comparisons’. Based on this matrix, we used the clustering methods to identify groups of homologous proteins that belonged to the same family or super-family.

Table 1 (top) shows the weighted F-measure, precision, recall and number of clusters (defined in Section‘Comparing the performance of the four sequence clustering methods’) obtained using the TransClust, HiFix, and MCL algorithms for protein family detection. The results obtained using SCPC were omitted because this method is more suitable for grouping super-family members. We observed that the performance of the other three methods degraded as the sequence similarity decreased. Indeed, all three methods produced the poorest performances on dataset A-10 (the most difficult case). The A-20 and A-30 datasets were also poorly clustered and none of the methods achieved a F-measure greater than 0.7. In general, TransClust, HiFix, and MCL did not produce performances over 0.77 on the ASTRAL datasets that contained many remote homologous proteins. On the other hand, the three methods efficiently and accurately clustered proteins in the GOLD dataset, which contains sequences with very high identity. TransClust outperformed the other three algorithms in terms of their general performance over all the datasets tested.

**Table 1:** **Sequence-sequence comparison F-measure for clustered sequences**

**Family**																
	**TransClust**				**HiFix**				**MCL**				**SCPS**			
**Dataset**	**F-measure**	**Clusters**	**Precision**	**Recall**	**F-measure**	**Clusters**	**Precision**	**Recall**	**F-measure**	**Clusters**	**Precision**	**Recall**	**F-measure**	**Clusters**	**Precision**	**Recall**
A-10	**0.494**	1757	0.834	0.409	0.467	2780	0.463	0.692	0.352	2310	0.923	0.389	-			
A-20	**0.573**	2013	0.885	0.494	0.491	3270	0.556	0.732	0.398	4125	0.999	0.278	-			
A-30	**0.675**	2561	0.912	0.628	0.583	3749	0.561	0.885	0.415	1827	0.351	0.773	-			
A-50	**0.721**	3221	0.903	0.709	0.608	4861	0.562	0.945	0.457	1912	0.702	0.445	-			
A-70	**0.739**	3486	0.904	0.733	0.630	4921	0.616	0.873	0.474	2323	0.752	0.482	-			
A-90	**0.758**	3630	0.913	0.753	0.653	4973	0.625	0.895	0.511	2824	0.815	0.512	-			
A-95	**0.766**	3715	0.916	0.765	0.654	4992	0.629	0.907	0.527	2873	0.527	0.813	-			
GOLD	**0.914**	96	0.905	0.968	0.902	99	0.960	0.895	0.880	56	0.808	0.942	-			
**Super-family**																
A-10	**0.377**	1757	0.917	0.281	**0.337**	2780	0.993	0.274	0.270	3270	0.997	0.180	0.297	658	0.387	0.221
A-20	**0.450**	2013	0.954	0.347	0.362	3270	0.993	0.293	0.282	4024	0.999	0.191	0.352	701	0.400	0.323
A-30	**0.551**	2561	0.551	0.440	0.473	3749	0.994	0.414	0.333	3745	0.998	0.235	0.473	792	0.494	0.364
A-50	**0.609**	3221	0.995	0.499	0.507	4861	0.992	0.457	0.351	3048	0.847	0.310	0.557	753	0.618	0.546
A-70	**0.631**	3486	0.997	0.519	0.539	4921	0.990	0.495	0.377	2086	0.875	0.335	0.581	493	0.649	0.518
A-90	**0.654**	3630	0.996	0.544	0.560	4973	0.989	0.528	0.426	2549	0.922	0.364	0.607	633	0.680	0.531
A-95	**0.659**	3715	0.996	0.552	0.563	4986	0.990	0.542	0.435	2616	0.912	0.378	0.615	940	0.686	0.542
GOLD	0.865	23	1	0.765	**0.915**	13	0.998	0.852	0.827	24	1	0.712	0.904	4	0.864	0.983

Number of clusters found, and weighted mean precision and recall values for each clustering algorithm are shown. Best values are shown in bold.

Table 1 (bottom) shows the performance of the four clustering methods for super-family detection. Overall, poorer F-measures were obtained for super-family detection compared with the values obtained for family detection. It is more difficult to group super-family members because, compared with a family, for a super-family group a common evolutionary origin cannot be deduced directly from sequence similarity. Therefore, clustering methods that use sequence-sequence similarity to form groups will not be able to detect distantly related proteins. As a consequence, the four methods produced poor performances over all the ASTRAL subsets; indeed, the F-measures were all less than 0.66 with TransClust producing the highest value. On the other hand, all four clustering methods achieved performances over 0.82 with the GOLD dataset, with HiFix producing the best performance.

### Profile-profile comparisons

**Figure 2:**
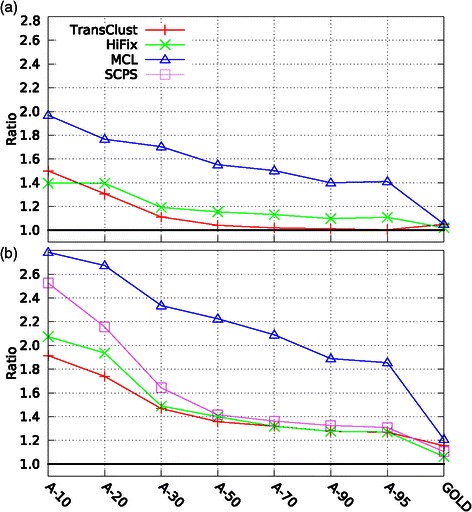
**F-measure improvement when using profile-profile comparisons.** Ratio between profile-profile comparison and sequence-sequence comparison F-measures for families **(a)** and super-families **(b)**.

**Figure 3:**
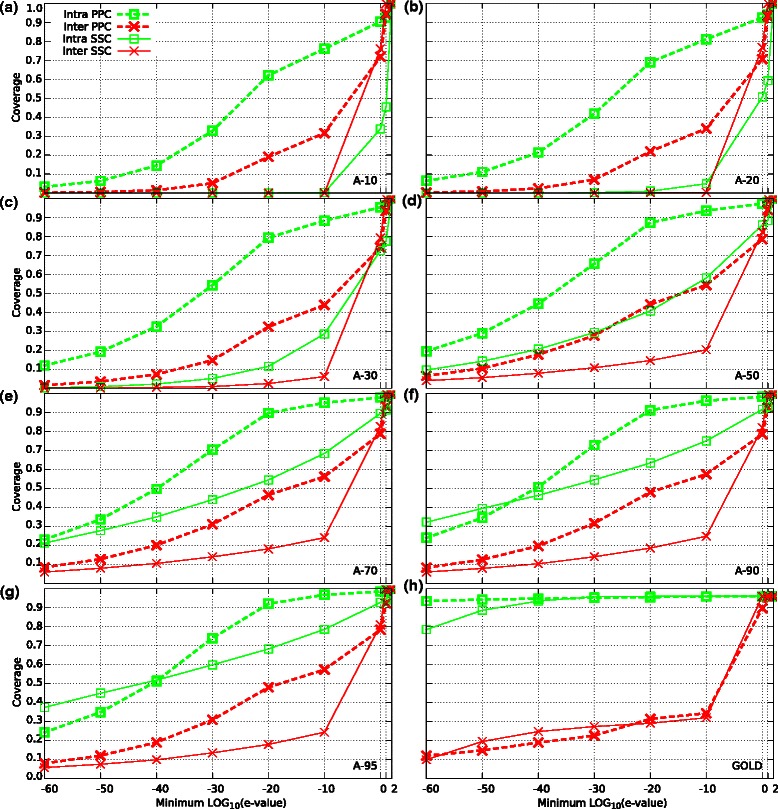
**Distribution of minimum e-values intra and inter families across all datasets.** Curves for Astral subsets A-10, A-20, A-30, A-50, A-70, A-90 and A-95 are showed in panels **a, b, c, d, e, f** and **g** respectively, and curves for Gold database is showed in panel **h**. E-values associated with sequence-sequence comparisons (SSCs) were computed by BLAST, while e-values related to profile-profile comparisons (PPCs) were obtained by combining HHBlits and HHsearch. For each protein in the datasets, we considered the e-value to the nearest neighbor from its own family (intra curves) and the e-value to the nearest neighbor from any other family (inter curves). Solid lines indicate BLAST e-values and dashed lines indicate HHsearch e-values.

**Figure 4:**
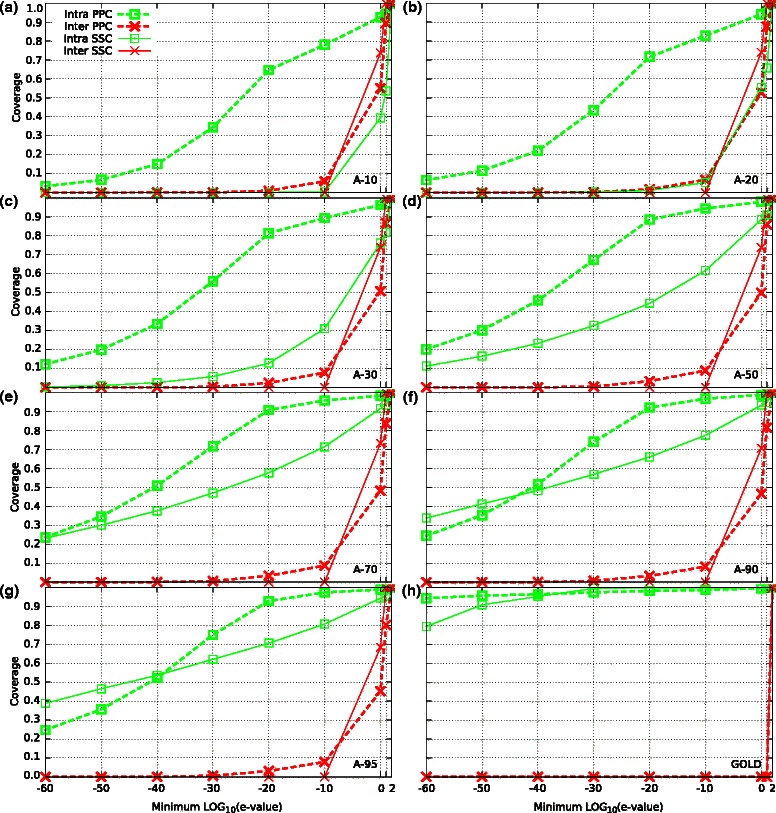
**Distribution of minimum e-values intra and inter super-families across all datasets.** Curves for Astral subsets A-10, A-20, A-30, A-50, A-70, A-90 and A-95 are showed in panels **a, b, c, d, e, f** and **g** respectively, and curves for Gold database is showed in panel **h**. E-values associated with sequence-sequence comparisons (SSCs) were computed by BLAST, while e-values related to profile-profile comparisons (PPCs) were obtained by combining HHBlits and HHsearch. For each protein in the datasets, we considered the e-value to the nearest neighbor from its own super-family (intra curves) and the e-value to the nearest neighbor from any other super-family (inter curves). Solid lines indicate BLAST e-values and dashed lines indicate HHsearch e-values.

To try to improve the performance of the clustering methods on remote homologous datasets, we construct a similarity matrix by extracting e-values from **P**rofile-**P**rofile **C**omparisons (PPCs), see Section ‘Profile–profile comparisons’. Using this new similarity measure, we found that the performance of all four clustering methods improved substantially across all the datasets for both family and super-family detection, see Table 2 and Figure 2a. The biggest improvement was obtained for the less similar dataset (A-10). A remarkable improvement was obtained in the clustering of super-family members (Figure 2b), showing that super-family detection can be improved using profile–profile comparisons. These results show that family and super-family detection depends crucially on the accuracy of the sequence searches and that search tools that embed evolutionary conserved properties produce better results than tools based on SSCs alone. It is interesting that the performance of the clustering methods with the GOLD dataset also improved for family and super-family detection. For super-family detection, all four methods properly clustered almost all members in the datasets, achieving F-measures of at least 0.97. Thus, even on datasets with very high sequence identity, PPCs provide a more accurate similarity measure than SSCs resulting in better family detection. These improvements were expected, once similarity measures based on PPC can better separate members of a given protein family/super-family (intra distances) from members of other families/super-families (inter distances), as visualized in Figures 3 (families) and 4 (super-families). Clearly, the areas between the PPC curves are larger than the areas between the SSC curves for most of the datasets in Figure 3 and for all datasets in Figure 4. We noted that the differences between the PPC and SSC areas were bigger for super-family than family detection. This finding reflects the larger improvements in super-family detection shown in Figure 2b.

**Table 2:** **Profile-profile comparison F-measure for clustered sequences**

**Family**																
	**TransClust**				**HiFix**				**MCL**				**SCPS**			
**Dataset**	**F-measure**	**Clusters**	**Precision**	**Recall**	**F-measure**	**Clusters**	**Precision**	**Recall**	**F-measure**	**Clusters**	**Precision**	**Recall**	**F-measure**	**Clusters**	**Precision**	**Recall**
A-10	**0.741**	1608	0.924	0.732	0.652	2590	0.648	0.916	0.693	783	0.730	0.653	-			
A-20	**0.749**	1773	0.912	0.760	0.685	3022	0.672	0.840	0.703	922	0.736	0.703	-			
A-30	**0.750**	2098	0.868	0.814	0.695	3147	0.678	0.899	0.707	1257	0.731	0.706	-			
A-50	**0.751**	2951	0.860	0.804	0.702	4534	0.702	0.900	0.709	1653	0.724	0.702	-			
A-70	**0.753**	3153	0.858	0.818	0.713	4673	0.709	0.909	0.712	1817	0.727	0.706	-			
A-90	**0.767**	2714	0.833	0.870	0.717	4708	0.889	0.710	0.715	1945	0.743	0.708	-			
A-95	**0.769**	2800	0.766	0.840	0.725	4725	0.709	0.907	0.743	2078	0.768	0.709	-			
GOLD	**0.959**	94	0.950	0.978	0.921	98	0.906	0.918	0.925	81	0.961	0.922	-			
**Super-family**																
A-10	0.722	1455	0.997	0.623	0.699	1182	0.963	0.636	**0.752**	714	0.908	0.726	0.750	186	0.742	0.763
A-20	**0.783**	1402	0.990	0.720	0.701	1319	0.964	0.644	0.754	848	0.916	0.738	0.759	253	0.934	0.654
A-30	**0.809**	1676	0.988	0.757	0.705	1500	0.942	0.686	0.778	1062	0.920	0.774	0.777	453	0.914	0.618
A-50	**0.827**	1995	0.987	0.778	0.710	2375	0.964	0.702	0.781	1642	0.968	0.723	0.789	665	0.958	0.693
A-70	**0.833**	2120	0.988	0.783	0.711	2476	0.960	0.707	0.788	1585	0.936	0.782	0.792	758	0.983	0.703
A-90	**0.835**	2213	0.988	0.779	0.715	2524	0.950	0.701	0.805	1799	0.965	0.755	0.805	931	0.993	0.700
A-95	**0.837**	2293	0.989	0.777	0.716	2582	0.960	0.708	0.807	1806	0.948	0.795	0.805	1023	0.995	0.703
GOLD	0.999	6	1	0.999	0.974	7	1	0.953	**1.000**	5	1	1	**1.000**	5	1	1

Number of clusters found, and weighted mean precision and recall values for each clustering algorithm are shown. Best values are shown in bold.

Comparing algorithms, TransClust produced the best performance with the PPC similarity measure, while the performance of MCL improved the most with the PPC measure.

### Parameter optimization

We tested 18371 proteins divided into eight datasets with the four clustering algorithms and two different clustering scenarios (family and super-family) by extensive experimentation and parameter variation. A total of 5776 experiments were performed. The parameters used in each clustering algorithm are given in parenthesis in Tables 3 and 4 and the range of parameters used is given in Table 5. Although there was a set of parameters for each clustering algorithm, most of time only one of the parameters drastically affected the results and this parameter was essentially related to the number of clusters obtained. For some of the algorithms, this special parameter was the initial threshold for sequence similarity that was used to group two proteins into the same cluster. Other algorithms such as MCL require information related to the density or the granularity expected in the clusters. For some algorithms, the maximum possible number of clusters has to be set like spectral clustering algorithms.

**Table 3:** **Sequence-sequence comparison F-measures for clustered sequences**

**Family**				
**Dataset**	**TransClust (** ***T*** **)**	**HiFix (** ***s,c*** **)**	**MCL (** ***I*** **)**	**SCPS (** ***c*** **)**
A-10	**0.494** (1)	0.467 (0.10,0.7)	0.352 (18)	-
A-20	**0.573** (1)	0.491 (0.15,0.7)	0.398 (17)	-
A-30	**0.675** (1)	0.583 (0.20,0.7)	0.415 (51)	-
A-50	**0.721** (1)	0.608 (0.25,0.7)	0.457 (40)	-
A-70	**0.739** (1)	0.630 (0.25,0.7)	0.474 (30)	-
A-90	**0.758** (1)	0.653 (0.25,0.7)	0.511 (29)	-
A-95	**0.766** (1)	0.654 (0.25,0.7)	0.527 (22)	-
GOLD	**0.914** (25)	0.902 (0.30,0.6)	0.880 (12)	-
**Super-family**				
A-10	**0.377** (1)	**0.337** (0.10,0.7)	0.270 (18)	0.297 (648)
A-20	**0.450** (1)	0.362 (0.10,0.7)	0.282 (18)	0.352 (753)
A-30	**0.551** (1)	0.473 (0.10,0.7)	0.333 (57)	0.473 (955)
A-50	**0.609** (1)	0.507 (0.25,0.7)	0.351 (59)	0.557 (1188)
A-70	**0.631** (1)	0.539 (0.25,0.7)	0.377 (43)	0.581 (1279)
A-90	**0.654** (1)	0.560 (0.25,0.7)	0.426 (43)	0.607 (1345)
A-95	**0.659** (1)	0.563 (0.25,0.7)	0.435 (28)	0.615 (1401)
GOLD	0.865 (1)	**0.915** (0.05,0.3)	0.827 (36)	0.904 (6)

The optimized set of parameters determined for each clustering algorithm are shown in parenthesis, see Section ‘Parameter optimization’. Best values are shown in bold.

**Table 4:** **Profile-profile comparison F-measures for clustered sequences**

**Family**				
**Dataset**	**TransClust (** ***T*** **)**	**HiFix (** ***s,c*** **)**	**MCL (** ***I*** **)**	**SCPS (** ***c*** **)**
A-10	**0.741** (15)	0.652 (0.10,0.7)	0.693 (26)	-
A-20	**0.749** (15)	0.685 (0.15,0.7)	0.703 (24)	-
A-30	**0.750** (15)	0.695 (0.15,0.7)	0.707 (24)	-
A-50	**0.751** (20)	0.702 (0.20,0.7)	0.709 (18)	-
A-70	**0.753** (20)	0.713 (0.20,0.6)	0.712 (17)	-
A-90	**0.767** (15)	0.717 (0.20,0.6)	0.715 (17)	-
A-95	**0.769** (15)	0.725 (0.20,0.7)	0.743 (17)	-
GOLD	**0.959** (50)	0.921 (0.30,0.6)	0.925 (15)	-
**Super-family**				
A-10	0.722 (1)	0.699 (0.10,0.6)	**0.752** (59)	0.750 (648)
A-20	**0.783** (5)	0.701 (0.10,0.7)	0.754 (59)	0.759 (753)
A-30	**0.809** (5)	0.705 (0.10,0.7)	0.778 (59)	0.777 (955)
A-50	**0.827** (5)	0.710 (0.15,0.7)	0.781 (58)	0.789 (1188)
A-70	**0.833** (5)	0.711 (0.15,0.7)	0.788 (60)	0.792 (1279)
A-90	**0.835** (5)	0.715 (0.15,0.7)	0.805 (59)	0.805 (1345)
A-95	**0.837** (5)	0.716 (0.15,0.7)	0.807 (60)	0.805 (1401)
GOLD	0.999 (1)	0.974 (0.05,0.5)	**1.000** (60)	**1.000** (6)

The optimized set of parameters determined for each clustering algorithm are shown in parenthesis, see Section ‘Parameter optimization’. Best values are shown in bold.

**Table 5:** **Variation range used to optimize the cluster parameters**

**TransClust**			**HiFix**	**MCL**	**SCPS**
*tmin*	*tmax*	*sz*	*s* and *c*	*I*	*c*
0.01	100	0.5	0.01, 0.05, 0.1,…, 0.9	0.1, 0.2,…, 6.0	90%, 95%,…, 120%

TransClust uses a similarity threshold to group proteins in the same cluster. We varied the similarity threshold *T* from 0.01 to 100 (*tmin* and *tmax* parameters). *T* directly affects the number of clusters reported because it is related to the similarity between members of the same cluster (i.e., lower values of *T* may lead to a small number of clusters).

To vary this parameter, a simple approach is to find the biggest gap or the most abrupt decrease in the frequency distribution of the e-values and set *tmin* and *tmax* to encompass this gap. The gap interval e-values have to be normalized by TransClust to obtain the proper *tmin* and *tmax* values. Another important parameter in TransClust is the *step size* (*sz* parameter), which is the value used to increase *T* from *tmin* to *tmax*. We set *s* *z*=0.5 because we found that values smaller than 0.5 produced the same results, and larger values are not advisable because the optimal *T* may be missed. Gradual variation of *T* will always yield the best results, although it increases the computation time.

For the MCL experiments, we varied the *inflation* (*I* parameter) from 0.1 to 6.0 in increments of 0.1, which is the maximum interval suggested in the program’s help file. *I* affects the granularity of the clusters that are produced; that is, *I* is related to the number of clusters obtained and cannot be determined directly by the frequency distribution of the e-values as was done for TransClust. To overcome this problem, a common approach is to use a validation subset (a dataset for which the correct family/super-family clusters are known) and to determine the *I* that produces the best performance.

For SCPS, the maximum number of clusters (*c* parameter) were varied from 90% to 120% (in increments of 5%) of the real number of families/super-families in the tested datasets.

A natural choice for *c* would be the size of the dataset (number of proteins), but this would be time-consuming and may result in one protein per cluster, which is rarely a realistic clustering solution. Therefore, we used only the *c* values described above. Moreover, we found that for *c*>110*%* the performance of SCPS showed no improvement.

For HiFix, the *sequence identity* (*s* parameter) and the *coverage* (*c* parameter) were varied from 0.01 to 0.9 in increments of 0.05. These parameters are used to connect two proteins in the similarity network if their sequence identity is greater than *s* and alignment coverage greater than *c*. These parameters are the same as the those used in the BLAST program. HiFix use *s* and *c* as filter constraints to select BLAST hits. Therefore, the same criteria used to set the sequence identity and the alignment coverage in BLAST can also be used to set *s* and *c*. Although *s* and *c* are used only to initiate the similarity network, often the quality of HiFix clusters is related to these parameters; that is, lower values will produce fewer clusters and higher values can result in singleton clusters (one protein per cluster).

### Practical usage

Here, we provide guidelines for the practical application of clustering sequence methods and discuss how our findings can help to detect homology relationships more accurately. We have focused the discussion on three applications: protein function prediction, comparative genomics, and construction of protein family databases. For each application, we discuss how clustering methods could be employed and how the methods can be improved using profile–profile comparisons.

#### Protein function prediction

Understanding protein functions is essential for comprehending the complex cellular machinery of living organisms. Functional characterization of newly discovered proteins is often performed by scanning databases for proteins that share sequence similarities with the new protein. Sequence similarity may suggest a common evolutionary origin between known function proteins and newly discovered ones. Thus, the new protein is annotated by transferring the annotation from closely related proteins. Clustering methods have also been used for protein function prediction, because proteins in the same cluster are likely to share the same function [9,10]. As input, clustering methods require a list of possible homologous sequences that can be obtained by comparing the target sequence to a database of annotated proteins, as described above. Next, a profile can be built for each sequence (target and annotated proteins) to allow the construction of a distance matrix based on profile-profile comparisons. This matrix can then be used as input for clustering methods that find groups of related proteins. The new protein can then be annotated by transferring the annotations of its nearest-neighbors.

#### Comparative genomics

Comparative genomics is a powerful tool for studying evolutionary changes among different species. The main goal is to identify genes/proteins that are conserved or common among different species, as well as those that are organism-specific. Indeed, comparative genomics studies have shown that every major taxonomic lineage contains a fraction of genes/proteins that lack recognizable homologs [11]. However, it is crucial to know whether a gene/protein is truly specific to a given genome or whether this absence is caused by technical limitations of the approach used to detect it. BLAST is frequently used to detect homologies in comparative genomics analysis; however, it is possible that some genes/proteins have diverged beyond the point at which they can be detected by the BLAST algorithms. Thus clustering methods that use profile–profile comparisons could be used to try to estimate the real number of organism-specific proteins in a given genome. Those methods could then identify connections between protein families that otherwise would have been missed by simple sequence comparisons.

#### Construction of protein family databases

Protein family databases are repositories of protein sequences organized according to one of the following criteria: their evolutionary relationships, their structural properties (e.g., structural classes, folds, 3D-motifs, and topology), or their sequence patterns (functional domains and motifs). Most protein family databases employ automatic clustering algorithms to group homologous proteins into families that are then analyzed manually by curators. The PPC methodology proposed here could be used to improve the quality of clusters detected automatically, thereby reducing the laborious work of experts.

## Conclusion

We measured the performance of four graph-based algorithms in clustering homologous protein sequences. In our analysis, we used several datasets with different degrees of sequence similarity to evaluate the capability of clustering methods in detecting remote homologous proteins, where homology relationships may not be apparent from sequence similarities alone. To group proteins, clustering methods need to establish a similarity measure to evaluate the closeness of a protein pair. This measure is directly related to quality and to the number of clusters produced by each algorithm. Generally, the similarity measure is derived from pairwise sequence alignments obtained from an all-against-all comparison with BLAST. However, pairwise sequence alignments do not produce an appropriate similarity measure for related proteins with very low sequence identity, as showed in Table 1. To circumvent these limitations, we have proposed a new similarity measure based on PPCs using HHblits and HHsearch (see Section ‘Profile–profile comparisons’).

We carried out extensive experiments using the standard SSC and the new PPC measures to evaluate the performance of clustering methods. Our experimental results show that the PPCs outperform the SSCs in measuring the similarity of highly divergent proteins. The new PPC measure improved the performance of all four clustering methods over all the datasets tested, as reported in Table 2. The improvements shown in Figure 2 were expected because it is well known that profiles capture evolutionary conserved properties in a set of related proteins that cannot be easily detected by pairwise sequence alignments. For large datasets, profile construction can be a time-consuming task; however, this task can be run in parallel executions.

Clustering algorithms have parameters that can be tuned to produce better results. We performed a parameter optimization step to determine the best configuration for each clustering method. We observed that the choice of these parameters is often completely empirical and the default parameters (sometimes suggested by the software packages) do not always produce the best results. As discussed above, these parameters can be determined by intrinsic information in the dataset (when it can be computed) or by a cross validation approach [12].

This work provides guidelines for the practical application of clustering sequence methods aimed at detecting homology relationships accurately. The resulting clusters constitute a useful information source for predicting the function and evolution of proteins.

We also envisage a number of new applications for the novel PPC similarity measure. For instance, the measure can be used to improve the clustering of protein complexes, accurately detecting protein-protein interactions [13].

## Methods

### Datasets

Two distinct datasets were used in this study. One dataset was based on subsets from SCOP (Structural Classification of Proteins) [3], and was used to measure the performance of sequence clustering algorithms on distantly related homologous proteins.

SCOP classifies all protein domains of known structure into a hierarchy with four levels: class, fold, super-family, and family. In this work, we used version 1.75 and considered the family and super-family levels. A SCOP family groups proteins with a clear evolutionary relationship, while a super-family groups families for which a common evolutionary origin was not obvious from sequence identity but are deemed probable based on an analysis of structure and from functional features. Because the SCOP dataset contains many redundant domains, we used the ASTRAL repository [14] to select non-redundant subsets. We extracted seven subsets of proteins from ASTRAL over a range of sequence identity thresholds and named them Astral-95 (A-95), Astral-90 (A-90), Astral-70 (A-70), Astral-50 (A-50), Astral-30 (A-30), Astral-20 (A-20), and Astral-10 (A-10), where A-*x* indicates that any pair of sequences in that subset shared at most *x*% sequence identity. We removed all singleton family/super-family from each subset so that there are no families or super-families with only one protein sequence. This step was necessary because singletons constitute an important percentage of the original ASTRAL dataset and may have subverted the results. For instance, 75% of families in A-10 were singletons; thus, if a clustering method created one cluster for each protein in A-10, it would have correctly group 75% of the dataset. Our aim was to evaluate the performance of clustering algorithms in grouping at least two remote homologous proteins into the same cluster.

The other dataset, GOLD [2], is a standard collection of homologous proteins and has been widely used to evaluate the performance of sequence clustering methods. The GOLD dataset contains 866 enzymes that were assigned manually to 91 protein families and five super-families. We included this dataset to show that sequence-clustering methods perform over 0.88 of F-measure when the homologous proteins in the dataset have high identity. Table 6 lists the number of sequences, families, and super-families in the ASTRAL subsets and GOLD dataset. Note that we did not remove singletons from the GOLD dataset because we wished to reproduce experiments that have been reported previously [5,7,15].

**Table 6:** **Number of sequences, families, and super-families in the datasets**

**Dataset**	**Sequences**		**Families**		**Super-families**	
A-10	3461	(55%)	970	(25%)	589	(30%)
A-20	4260	(60%)	1144	(28%)	684	(34%)
A-30	6532	(72%)	1572	(38%)	868	(44%)
A-50	10816	(84%)	2109	(49%)	1080	(55%)
A-70	13391	(87%)	2306	(54%)	1162	(59%)
A-90	15861	(90%)	2420	(56%)	1222	(62%)
A-95	17505	(91%)	2521	(59%)	1273	(64%)
GOLD	866	(100%)	91	(100%)	5	(100%)

Numbers in parenthesis indicate the percentage of sequences/families/super-families that remained after removing singletons.

### Similarity measures

A crucial step in the application of clustering algorithms is how to measure the similarity between a pair of proteins. This measure is used by clustering algorithms to form clusters of homologous proteins. Most methods perform an all-against-all BLAST comparison of a given dataset and then use e-values or percentage of sequence identity as a distance measure between two sequences. However, more sensitive sequence comparison methods that uses multiple sequence alignments represented as Hidden Markov Models or sequence profiles have been proposed [16]. In this study, we investigated whether similarity measures based on PPCs yielded better results than the standard SSC similarity measure.

#### Sequence–sequence comparisons

BLAST is a program that is widely used to compare protein sequences. It performs pairwise sequence alignments using a heuristic approach that locates short matches between a pair of sequences rather than comparing whole sequences. The heuristic algorithm makes BLAST much faster than other methods that calculate optimal alignments. For this reason, it is commonly used in clustering sequence methods to provide sequence similarities.

Most of the currently available sequence clustering methods use e-values as the similarity measure rather than sequence identities. BLAST outputs can display several hits for a pair of sequences and their e-values can differ. Clustering algorithms that use e-values make some assumptions: 1) uniqueness (only one similarity value for a given protein pair), and 2) symmetry (the similarity between two proteins *i* and *j* is equal to the similarity between *j* and *i*). Thus, e-values must be pre-processed before being used as similarity measures. Typically, the highest, lowest, or average value of hits for an aligned protein pair are used [5].

For each dataset, we conducted an all-against-all BLAST search with a permissive e-value threshold of 100 and with all other parameters as default values [17]. The BLAST output was then used as input for each clustering algorithm that transformed e-values into unique and symmetric similarity measures before using them to form groups. Details about e-values transformation can be found in the original papers (see Section ‘Clustering approaches’).

#### Profile–profile comparisons

Instead of comparing individual protein sequences, PPC tools [16] compare evolutionary patterns encoded in a profile Hidden Markov Model (pHMM) [18]. A pHMM is a probabilistic model built from a set of related aligned sequences. The model describes common physicochemical and evolutionary properties shared by a group of proteins. Clearly, a profile can incorporate more information about the conservation and evolution of individual positions or segments within a protein than the amino acid sequence alone. Because this information (dictated by structure and function) is often better captured in a profile than in a single sequence, profiles can help to detect similarities between homologous proteins that are very diverged. Consequently, PPCs represent a more sensitive strategy than SSCs for detecting distant evolutionary relationships among proteins [19].

Here, we investigated whether similarity measures based on PPCs could yield better protein clusters than the standard SSCs. We used two tools from the HHsuite package [20]: HHblits [8] and HHsearch [16]. HHblits is an iterative method that builds a profile for a query sequence from a special profile database (built from the UniProt and NCBI nr databases and provided in the HHsuite package), and HHsearch compares profiles (it takes a profile and searches for similar ones in a profile library). First, we used HHblits (with default parameters) to construct a profile for each sequence in our datasets. Then, we conducted an all-against-all profile–profile search using HHsearch (with default parameters), which provided e-values similar to what BLAST does. Because most sequence clustering algorithms cannot interpret HHsearch outputs, we converted them to match the output format used by BLAST. The script that we used for this transformation is available for download (see Section “Availability”).

### Clustering approaches

The four algorithms that we assessed are based on graph-structure clustering methods that use a graph to represent the protein space, where proteins are modeled as vertices and the weight of an edge connecting two proteins corresponds to their similarity, which is normally extracted from SSCs (see Section ‘Sequence–sequence comparisons’). or alternatively from PPC (see Section ‘Profile–profile comparisons’). Although the algorithms themselves are not the focus of this paper, we describe them briefly below.

#### Transitivity clustering

TransClust [5] (short for transitivity clustering) uses an approach that is based on graph modifications. It is generally defined as the weighted transitive graph projection problem (WTGPP) [21], which consists of transforming a given intransitive graph into a transitive one by adding or removing edges. A transitive graph is a set of sub-graphs completely connected, where different sub-graphs are not connected by an edge (a disjoint union of cliques). The clusterization process begins by connecting two proteins *i* and *j* by an edge (*ij*) if their similarity (*s*(*i* *j*)≥0) exceeds a user-given *threshold* (*T*≥0). Otherwise, if *s*(*i* *j*)≤*T*, *i* and *j* are not connected by an edge. Next, TransClust iteratively adds and removes edges from the obtained graph, transforming it into a disjoint union of cliques. Each edge addition or removal has a cost, and the sum of all costs (which is the objective function to be minimized) cannot exceed an upper bound *C*. For instance, edge *ij* has deletion cost *s*(*i* *j*) if proteins *i* and *j* were joined by an edge or addition cost *s*(*i* *j*) otherwise. In other words, the cost function penalizes the removal of edges with a very high sequence identity and the addition of edges with low identity.

An optimal solution for WTGPP is NP-hard [22], meaning it cannot be found in a polynomial-time. Therefore, TransClust adopts a combination of heuristic and exact methods to find a close to optimal solution in reasonable time. First, it utilizes CAST (Cluster Affinity Search Technique) [23], a greedy heuristic, to estimate an upper bound of the emerging cost *C* to solve the WTGP problem. Depending on the estimated CAST cost, TransClust uses an exact fixed-parameter algorithm [21] or an improved version of the heuristic called FORCE [24]. Finally, the clustering solution with the lowest costs is reported.

#### Spectral clustering of protein sequences

Spectral clustering methods [17] exploit the eigenvalues and eigenvectors obtained from a similarity matrix (henceforth called *S*) to partition objects into disjoint clusters. Several spectral clustering implementations have been reported in the literature. Here, we evaluated one such method, Spectral Clustering of Protein Sequences (SCPS), proposed in [6].

SCPS first finds small clusters containing less than five proteins by carrying out a connected component analysis and eliminates rows and columns of the refereed proteins from *S*. This is done because it is unlikely that these proteins will be separated further during the next steps. Second, SCPS normalizes *S* producing *S* ^′^ and then calculates a matrix *L*=*D* ^−1/2^ *S* ^′^ *D* ^−1/2^, where $d_{\textit {ii}}=\sum _{j}\left (s^{'}_{\textit {ij}}\right)$ is an element of the diagonal matrix *D* and $s^{'}_{\textit {ij}}$ is the normalized similarity between proteins *i* and *j*. In *L*, the dissimilarities are generally greater than in *S*. Third, the columns of a matrix called *U* are obtained from the eigenvectors of *L* corresponding to the *K* largest eigenvalues of *L*. *U* is normalized such that each row sums to no more than one.

Finally, the rows in *U* are handled as points in the $\mathbb {R}^{K}$ dimensional space and the K-means algorithm [25] is used to group these points into *K* clusters. Note that in this new space each row in *U* represents a protein, and the number clusters *K* can be derived from the eigenvalues of *S* [17].

#### Markov clustering algorithm

The MCL (Markov cluster) algorithm [4] is based on the idea of random walks on graphs. A random walk is a path obtained by a succession of random steps through vertices of a weighted graph *G*. It can be used as a clustering strategy, because it is more probable that a random travel initiated at a vertex *i* of a cluster *C* leads to a vertex *j* in the same cluster than to a vertex outside *C*.

The MCL algorithm proceeds in the following manner. First, it obtains the weights of *G* edges by the transformation of similarities measures into probabilities. Next, it simulates a random walk in *G* and updates the probabilities of the obtained path. Thereby, MCL generally boosts the probabilities associated to edges within clusters and weakens those from different clusters. To perform the updates, MCL applies two operators: *inflation* and *expansion*. The *inflation* operator is responsible for increasing the probabilities of intra-clusters edges and decreasing those from inter-cluster edges during random walks. Accordingly, the *expansion* operator attenuates the probability of higher length paths. These two operators may be interpreted as opposite forces, because longer paths are expected within clusters rather than outside. Until an equilibrium is reached, MCL alternates between *inflation* and *expansion* and gradually updates some edge probabilities to zero, which has the same effect as eliminating these edges. Therefore, MCL will split *G* into a disjoint set of vertices or clusters.

#### HiFix

HiFix (High-Fidelity clustering of sequences) [7] exploits the topology of a similarity network (represented by a weight graph) and multiple sequence alignments to find groups of related proteins. HiFix uses SiLiX [15], an ultra fast clustering algorithm, to obtain pre-families that are later split into real families. SiLiX is an iteratively algorithm that performs a connected component analysis on a graph that represents the protein space. First, edges are examined to find trees containing both vertices as roots and then the resulting trees are merged to form a new tree. This step is repeated until all the trees are transformed into star trees for which the root is the most representative member of the family. These star trees constitute the clusters found by the SiLiX.

HiFix uses a pipeline divided into three steps. First, SiLiX is performed with low-stringency criteria to produce a few sets of large pre-families. These sets probably will have few false negatives, but possibly many false positives. Second, each pre-family set is decomposed into more homogeneous protein clusters using Lovain [26], an algorithm that can analyze the topology of a graph to find communities (clusters). Because homologous sequences belonging to the same protein family can be present in different communities, a third step is required to merge the communities predicted by Louvain. For that, HiFix evaluates each community on the basis of multiple alignment likelihood using its pHMM. For a set of sequences *P*={*p*_1_,…,*p*_*n*_} distributed into *Q* communities, HiFix performed *Q* multiple alignments to obtain the corresponding pHMMs that are used to compute the completed log-likelihood, which is used to evaluate the quality (homogeneity) of each cluster in *Q*. To merge communities, HiFix uses the connectivity measure *π*_*ql*_ computed by Louvain, where *π*_*ql*_ is the probability that vertices of different communities *q* and *l* are connected. Finally, HiFix progressively merges the two clusters with highest *π*_*ql*_ and computes the Integrate Classification Likelihood (ICL) [27] to measure the quality of all resulting clusters. This step is repeated until a single cluster is achieved, HiFix returns the set of clusters with the highest ICL.

### Comparing the performance of the four sequence clustering methods

Various quality measures have been proposed to evaluate the quality of a given clustering method. To the best of our knowledge, no quality measure optimally captures the notion of a natural cluster; i.e., a cluster that reflects a real group of common elements. Moreover, it is a well known that for each quality measure there is an example where it fails as a measure [28]. Also, no polynomial-time algorithm for the optimization of these measures is currently available. However, each quality measure captures at least one aspect of a natural cluster; therefore, these measures can be used as criteria for distinguishing efficient clustering methods from others that are less efficient.

Here, we used the F-measure to evaluate the performance of sequence clustering methods on the GOLD and ASTRAL datasets. The F-measure integrates both precision and recall and has been used widely to measure the correctness of clustering algorithms [6,7,21]. We first define precision and recall and then we describe how the F-measure is computed.

Let *n* be the total number of proteins in a given dataset, *n*_*f*_ the number of proteins within the *f*th family or super-family, *n*_*g*_ the number of proteins placed in the *g*th cluster, and *n*_*fg*_ the number of proteins shared by both the *f*th family/super-family and the *g*th cluster. The precision of cluster *g* with respect to *f*th family/super-family is then defined as *p*_*fg*_=*n*_*fg*_/*n*_*g*_. Precision measures the fraction of proteins in cluster *g* that are present in the *f*th family/super-family. In addition, the recall measures the fraction of proteins of the *f*th family/super-family detected by the *g*th cluster. Recall is given by *r*_*fg*_=*n*_*fg*_/*n*_*f*_. F-measure is a weighted harmonic mean between precision and recall given by $\frac {1}{n}\sum \limits _{f}{n_{f} \cdot \underset {g}{max} \frac {2 \cdot p_{\textit {fg}} \cdot r_{\textit {fg}}}{p_{\textit {fg}} + r_{\textit {fg}}}}$, where *n* is the total number of proteins in the dataset. Note that the weighted mean precision and recall are given by $\frac {1}{n}\sum \limits _{f}{n_{f} \cdot \underset {g}{max} \cdot p_{\textit {fg}}}$ and $\frac {1}{n}\sum \limits _{f}{n_{f} \cdot \underset {g}{max} \cdot r_{\textit {fg}}}$, respectively.

## Availability

All the datasets used in this study, along with the bash and Perl scripts are freely available at http://www.lcqb.upmc.fr/julianab/software/cluster/.
